# Approaching “highly sensitive person” as a cultural concept of distress: a case-study using the cultural formulation interview in patients with bipolar disorder

**DOI:** 10.3389/fpsyt.2023.1148646

**Published:** 2023-09-22

**Authors:** Michael Ioannou, Sofia Olsson, Ane Bakken Wold, Marzia Dellepiane, Steinn Steingrímsson

**Affiliations:** ^1^Institute of Neuroscience and Physiology, University of Gothenburg, Sahlgrenska Academy, Gothenburg, Sweden; ^2^Region Västra Götaland, Psykiatri Affektiva, Department of Psychiatry, Sahlgrenska University Hospital, Gothenburg, Sweden; ^3^Region Skåne, Office for Psychiatry and Habilitation, Psychiatric Clinic Lund, Lund, Sweden

**Keywords:** cultural formulation interview, highly sensitive person, bipolar disorder, DSM-5, cultural psychiatry, cultural concept of distress

## Abstract

**Background:**

Psychiatric patients may refer to concepts neither medically accepted nor easily understood to describe their experiences when seeking medical care. These concepts may lie outside the clinician’s cultural references and consequently hinder the diagnostic consultation. In the fifth version of the Diagnostic and Statistical Manual of Mental Disorders (DSM-5), the clinical instrument Cultural Formulation Interview (CFI) was included. The CFI aims to facilitate the gathering and synthesis of culturally relevant clinical information. The notion of Cultural Concepts of Distress (CCD) was also introduced in DSM-5. The CCD include the subterms of the cultural syndrome, cultural explanation, and cultural idiom of distress. No previous study has used CFI for conceptualizing a cultural notion as a CCD. This study aimed to approach the cultural notion of being a highly sensitive person (HSP) in patients with bipolar disorder (BD) by applying the CFI. The cultural notion of HSP has garnered great interest globally, although scientific evidence is limited. No direct correlation between BD and HSP was hypothesized before or during the study process.

**Methods:**

In this case study, three patients with BD who reported being HSP were interviewed using the CFI. Furthermore, the applicability of the CCD was examined based on the outcomes of the CFI using an interpretive approach.

**Results:**

All three patients reported that the CFI facilitated the clinical consultation, and in one of the cases, it may also have increased the treatment engagement. Based on the synthesis of the CFI outcomes in these illustrative cases, HSP could be understood as a cultural syndrome, a cultural explanation, and a cultural idiom of distress.

**Conclusion:**

By applying a person-centered perspective, CFI was used for the conceptualization of a cultural notion as a CCD (i.e., HSP in our study). Moreover, the cases highlight the complexity of illness insight in BD as a medical phenomenon when patients’ illness perspectives are taken into consideration. Future studies need to further examine the clinical relevance of the CFI in the management of BD.

## Introduction

A common clinical challenge in the encounter between patient and clinician is grasping the patient’s narrative and the importance of her lifeworld in order to elicit a deeper understanding (1). During the encounter between patient and clinician, the patient may use concepts outside of the clinician’s cultural references to explain their experiences. The clinicians may have difficulty grasping these concepts because they lack acceptance from the broader medical community (2). The difficulty in understanding these concepts may hinder the diagnostic process and jeopardize treatment adherence.

To address these challenges several changes were made in the fifth version of the Diagnostic and Statistical Manual of Mental Disorders (DSM-5). A major change was the inclusion of the clinical instrument Cultural Formulation Interview (CFI) to facilitate the gathering and synthesis of culturally relevant clinical information (3). The core CFI consists of 16 open-ended questions which cover four significant domains: cultural definition of the problem; cultural perceptions of cause, context, and support (including cultural identity); cultural factors that affect self-coping and past help-seeking behavior; cultural factors that affect current help-seeking behavior (4). Supplementary modules that expand on the above-mentioned domains of the 16-item version of CFI (i.e., core CFI) are also available (5). The CFI has been validated internationally in varied clinical settings (6, 7).

Another important change in the DSM-5 was the introduction of the Cultural Concepts of Distress (CCD) partly in response to the critique of the previous notion of culture-bounded syndromes (8). The CCD incorporate the terms of the cultural syndrome, cultural explanation, and cultural idiom of distress. These terms are not mutually exclusive but often overlap and interact with each other. The overlap among these terms reflects the complex and multifaceted nature of cultural influences on distress and mental health. However, there are conceptual differences between these terms as they highlight different aspects of how cultural groups may experience, understand, and communicate distress, behavioral problems, or troubling cognitions and emotions (9). *Cultural syndromes* are patterns of symptoms that are recognized within a particular cultural group but may not fit neatly into existing diagnostic categories. They can be approached as local distress categories based on layman’s theories which are supported by collective experience and anecdotal evidence. Thus, these syndromes reflect culturally specific ways of experiencing distress. *Cultural idioms of distress* refer to culturally specific expressions or phrases that people use to describe their distress. They can include metaphors, proverbs, or symbolic language that captures their emotional or psychological state. The CCD also encompasses the cultural beliefs and explanations about the causes, nature, and meaning of mental health problems. These explanations may involve religious, spiritual, or supernatural beliefs, as well as social and environmental factors. Hence, *cultural explanations of illness* provide a framework for understanding distress and guide help-seeking behaviors within a cultural context.

*Highly sensitive person* (HSP) is a Western concept coined by the American psychologist Elaine Aron in the late nineties (10). It is based on a theory of innate traits of higher receptivity to both internal and external stimuli, such as emotional, environmental, and social triggers (i.e., sensory processing sensitivity) (11, 12). High sensitivity is commonly assessed using a self-report scale on HSP traits without the requirement of professional assessment (13). The construct of HSP is related to and possibly conflated with introversion, neuroticism, and narcissism (14–16). Sensory processing difficulties may be a general pattern in several psychiatric disorders without an apparent connection to certain psychopathology (17).

From a scientific perspective, the concept of HSP has been criticized as diffuse with questionable validity and unclear distinction from other established psychological constructs (16, 18). From a philosophical viewpoint, HSP has been described as an example of the medicalization of human suffering (19). Suffering is namely considered to be an integral part of being a human person. Where previously philosophy, politics, and religion provided insights about how to understand and deal with this suffering, nowadays the interpretive authority is medical science (19).

Although the empirical knowledge on HSP is still limited, societal interest has been very high (11). Several online communities, popular science books, and self-help guides are available in various countries and cultures. Moreover, there are several online advertisements for therapists who report to be specialists in HSP (20–22). Individuals who self-identify strongly with HSP describe both positive and negative experiences related to this trait (16). Deep emotional empathy and strong ability to recognize other perspectives are usually described as positive features although they may lead to stress, exhaustion, and overstimulation (16, 23). The self-attribution of HSP may lead to other positive features such as self-acceptance, the sense of belonging to a community and liberation from the feeling of being deficient or an outsider (16). Sociological studies have reported similarities between the discourse of HSP (as a sociocultural phenomenon) and the disability activism related to neuropsychiatric diagnoses. Namely, HSP communities have been formed to counteract the negative preconceived notions about HSP, highlight their needs in society and produce knowledge in their own terms (24–26). In sum, the concept of HSP has received great attention in everyday psychology beyond its scientific foundation and several individuals self-label themselves as HSP. Hence, psychiatric patients can also report HSP-traits and it may lead to challenging clinical encounters.

A cultural psychiatric perspective could enhance the clinical understanding of patients who report HSP. By embracing a cultural psychiatric perspective, the patient’s reported distress is grasped firstly as an act of communication so as to understand its intended meaning in the light of the patient’s sociocultural background before engaging in person-centered clinical reasoning (27). Such an approach may also provide a meaningful framework to discuss how cultural concepts are related to medically defined severe mental disorders. Furthermore, previous studies have mainly focused on the encounter of Western-trained clinicians with patients with non-western cultural schemas or scripts. Hence, there has been little focus in the literature on Western cultural concepts within the Western psychiatric setting.

In this study, we aimed to approach the notion of HSP among three patients with the mental disorder of bipolar disorder (BD) utilizing the CFI and testing the relevance of the CCD in a real-life clinical setting. BD is a severe chronic mental disorder with a high burden of disease and excess mortality (28, 29). The clinical manifestation of BD is characterized by changes in a person’s mood, energy, and ability to function, formally experienced as intense emotional states during distinct periods (i.e., mood episodes of mania or depression) (30). In addition, changes in illness insight and self-awareness may also occur, which is often associated with pharmacological noncompliance and poor treatment outcomes (31).

## Methods

This case study is based on three patients with a previously confirmed diagnosis of BD who reported HSP. The patient recruitment took place at the psychiatric department of the Sahlgrenska University Hospital in Gothenburg, Sweden. The three patients were interviewed by their clinician (MI) using the core CFI as part of the clinical consultation. The collected data were discussed and analyzed with other colleagues (SS, SO, MD) as a part of an internal educational program on cultural competence. The interpretation and synthesis of the CFI outcomes were based on the theoretical framework of the cultural material available in the DSM-5 (9). No other systematic qualitative data analysis was conducted. Furthermore, no direct correlation between BD and HSP was hypothesized before or during the study process. The presented cases are anonymized by removing personally identifiable data while retaining the value of the presented information. Before interviewing, the patients have given orally their consent to be described in case reports. This study is part of a larger project approved by the Swedish Ethical Review Authority (DNR 2020–05807). The overall project is an explorative retrospective medical chart review study for identifying and evaluating relevant factors to the treatment outcome in patients who have been hospitalized for affective disorders. Parts of the study were previously presented at the 25^th^ European Congress of Psychiatry (32). The Swedish version of the core CFI was used, without CFI supplements, in line with previous studies (33, 34).

## Results

The three cases are presented below. The patients were of Swedish origin or second-generation immigrants with Swedish as their native language. All three patients reported that the CFI facilitated the clinical consultation by eliciting a person-centered approach. In the second case, the use of CFI positively affected the treatment engagement. However, there was no reported change in treatment engagement in the first or the third case.

### Case 1: HSP as a cultural syndrome

A woman in her mid-twenties was referred to an outpatient clinic for a post-discharge appointment after hospital admission. She was diagnosed with BD type I during the hospital admission. The visit occurred two weeks after the involuntary admission for a manic episode with psychotic features. Lithium treatment had been initiated during the hospital admission as a long-term prophylaxis of new mood episodes. However, the patient had discontinued lithium after the hospital discharge but wanted to come to the follow-up visit to the outpatient clinic. The CFI was conducted as part of the first outpatient consultation. She wasn’t interested in taking any medication but curious about the outcomes of the diagnostic investigation. She had doubts about BD and psychiatry in general. Other family members had been diagnosed with BD, but she could not see any similarities between their behavioral patterns and her own. However, her family members’ experiences and stories negatively affected her expectations from psychiatry. She referred to herself as being a HSP who needed to take it easy. Many persons from her social network happened to identify as HSP. She felt well-understood and supported by the community of HSP. Indeed, she explained her hospital admission as necessary after a mental breakdown due to her high sensitivity. Stimulus reduction was namely a significant measure for her recovery, according to her. After two more follow-up visits, the patient discontinued her contact with the outpatient clinic.

For this patient, HSP was an important part of her cultural identity. She gave meaning and understanding to her experienced distress through the lens of the collective experience of HSP. Self-identifying as HSP was incompatible with also having a mental disorder. The self-attribution of HSP was far more than communicating or explaining her distress. The concept of the cultural syndrome was found to conceptualize her narrative better than the terms of the cultural idiom of distress and cultural explanation.

### Case 2: HSP as a cultural explanation

A man in his mid-fifties was admitted to the psychiatric hospital due to bipolar depression with psychotic symptoms. He had been diagnosed with BD type I two decades ago and had been on a long-term lithium treatment since then. He had a good treatment response to lithium. However, a few months before the psychiatric admission, he interrupted his lithium treatment and discontinued his contact with the outpatient clinic after reading a book on HSP. The CFI took place during the last phase of the hospital admission, where the patient gradually improved, but he was still unwilling to restart with lithium treatment. During the CFI, he could describe in detail how the HSP concept explained many of his psychiatric symptoms. He regarded HSP as the main cause of his bipolarity, and to find balance in his own life, he needed to engage with that sensitivity actively. During the interview, the patient argued for his theory of the causes behind his chronic mental health issues, and he was convinced that he needed to continue adjusting his life based on the general recommendations for HSP. The clinician tried to bridge the differences between the explanatory models (i.e., HSP and the medical model of BD). At the end of the interview, the patient accepted lithium as a complementary therapy in his HSP-related lifestyle interventions. The patient continued with lithium treatment and has been without mood episodes for at least two years.

The patient, although skeptical of the psychopharmacological treatment, did not deny the relevance of the psychiatric diagnosis of BD. It remained uncertain to which extent he could accept a biomedical explanatory model of his mental distress except for his self-diagnosed genetic predisposition to sensory sensitivity. In a sense, HSP was perceived as the underlying pathophysiologic mechanism of his mood states. The concept of cultural explanation could conceptualize adequately the use of HSP in his illness narrative.

### Case 3: HSP as a cultural idiom of distress

A woman in her late thirties diagnosed with BD type II was interviewed with the CFI during an annual check-up visit at an outpatient clinic. During the CFI, she described how she experienced and communicated her distress to others. She found the diagnosis of BD stigmatizing and often a barrier to seeking understanding and support from her network. She preferred to describe herself as “highly sensitive” rather than “bipolar” or “vulnerable,” as her disorder was not all about distress. Her sensitivity could be a source of creativity, sympathy, and emotionality. She appreciated that the clinician was interested to know more about her interpretation and management of her disorder. If doctors wanted to refer to her as “bipolar,” she would accept it as long as it did not leave the room. She already had good medication compliance, which did not change after the CFI assessment.

In this case, the concept of the cultural idiom of distress was found relevant when approaching her illness narrative. The patient showed a similar understanding of her illness as her psychiatrist, without a report of parallel explanatory models based on HSP traits. The patient had good adherence to treatment without any change after the use of CFI.

## Discussion

In this study, we argue that the concept of HSP can be approached as a CCD. This argument is based on the synthesis of the CFI outcomes in the illustrative cases described above, where HSP could be understood as a cultural syndrome, a cultural explanation and as a cultural idiom of distress. To our knowledge, this is the first study using the clinical instrument of CFI to conceptualize a cultural notion as a CCD. Furthermore, this study demonstrates the potential clinical usefulness of CFI and the relevance of CCD, even when encountering patients with Western cultural representations. Lastly, this study also comments on the ongoing ¨looping effect¨ (i.e., reciprocal interplay) between science and local concepts of illness (9, 35).

It has been suggested that several conditions need to be in place for a cultural representation to be popularized (36, 37). Indeed, the case of HSP may fulfill several of the following conditions: credible and prestigious source (e.g., peer-reviewed publications); moderate levels of novelty; well fitted with other available representations (such stress and copying theories or the medical model of BD); easy to communicate; strong emotional appeal and psychological ¨susceptibility¨ of people who learn these ideas and pass them (for instance, patients defensiveness toward psychiatric reasoning, as illustrated in our cases). However, several studies are needed to strengthen this argument further.

It must be emphasized that we do not argue for a direct correlation between BD and HSP. The aim of the study was neither to examine the validity of HSP as a clinical phenotype nor its utility as a diagnostic concept. However, the relevance of emotional processing in BD has previously been discussed in the literature (38–41). In this regard, sensory processing sensitivity may prove to be relevant in the pathophysiology of BD and is worthy of further study.

Furthermore, the presented cases highlight the complexity of illness insight in BD as a medical phenomenon when patients’ illness perspectives are taken into consideration. Indeed, illness insight is a well-debated topic, and it may be understood better as a continuous, dynamic, and multidimensional concept (42, 43). Moreover, patients with BD may show a great awareness of disease features but poor insight into their own mental state (44). Interestingly, the level of illness insight is state dependent with good recovery of insight during periods of remission (45). However, traditional medical understanding of insight often does not consider lay perceptions of illness, and other explanatory frameworks (46, 47). This may lead to a fragmented or myopic perspective of a person’s illness insight. Hence, a narrative approach has been suggested emphasizing the subjective dimensions of insight, its process formation and intended effects in a specific socio-cultural context (48, 49).

This case-study has some limitations. The main conclusions are based on the synthesis of the CFI outcomes in three selected patients without applying a systematic case study analysis. Using this approach, a hypothesis may be generated but not examined in depth (50). Moreover, the data were interpreted using the theoretical ground established on the cultural material of DSM-5 without other in-depth analyses taking place. Hence, the typification of CCD may be considered arbitrary. However, there is no clear consensus on the proper research methodology for the investigation of CCD and psychiatric disorders (51). A possible way forward is to further sharpen the conceptualization and definition of CCD and distinguish the subterms from each other.

Future studies need to further examine the clinical relevance of CFI in the management of BD. Three directions may be of interest. The first direction regards the diagnostic accuracy of BD. The diagnostic reliability of BD may be vulnerable from a sociocultural perspective (52, 53). Biases in the diagnostic assessment have previously been explained by racial bias (54), linguistic and vocabulary problems (52), misattribution of psychotic symptoms (54, 55), different perceptions of mania severity (56), and relevance of life events in mood alterations (57, 58). Proper integration of the CFI in the diagnostic process may resolve many of these issues. Indeed, a recent study found that CFI may facilitate the identification of symptoms of certain psychiatric disorders among non-native-speaking patients in a migration context (33). The second direction considers the assessment of patients’ perception and illness insight in BD. Combined with established psychometric instruments, CFI could facilitate a broader assessment of insight in BD. And thirdly, CFI could contribute to a more person-centered approach in the clinical encounter with patients with BD. Thus, personal meaning and narratives will not be overwritten but rather highlighted. By having a shared narrative as a starting point, the collaboration in setting goals and disease management could be facilitated, leading potentially to better long-term outcomes for the patient.

## Data availability statement

The original contributions presented in the study are included in the article/supplementary material. The data collected in the study are presented as fully as possible without revealing individual identity and no additional data is available.

## Ethics statement

The studies involving human participants were reviewed and approved by Swedish Ethical Review Authority, Sweden. Written informed consent for participation was not required for this study in accordance with the national legislation and the institutional requirements.

## Author contributions

MI and SS were involved in the conceptualization of the study. MI conducted the interviews, undertook administration of the project, and wrote the first draft of the article. SS supervised the study. All authors were involved in the review, and editing of the article, and approved the submitted version.
